# The Mediating Effect of Innovation Self‐Efficacy and Organizational Innovation Atmosphere on Digital Leadership and Green Creativity of Clinical Nurses: A Cross‐Sectional Study

**DOI:** 10.1155/jonm/9733332

**Published:** 2026-04-07

**Authors:** Yu Han, Yanmei Wang, Yan Liu, Zezhou Wang, Xue Dong, Xiaoxuan Zhao, Xinyu Liu, Kui Fang

**Affiliations:** ^1^ Department of Humanistic Nursing, School of Nursing, China Medical University, No.77 Puhe Road Shenyang North New Area, Shenyang, 110122, Liaoning, China, cmu.edu.tw; ^2^ Department of Neurosurgery, The First Affiliated Hospital of China Medical University, Shenyang, China, cmu.edu.cn

**Keywords:** digital leadership, green creativity, innovative self-efficacy, nursing management, organizational innovation atmosphere, sustainable development

## Abstract

**Background:**

The healthcare industry is facing trends toward digital transformation and sustainable development. Green creativity, defined as nurses’ ability to propose innovative environmental solutions in clinical practice, has become an important driving force for the transformation toward green healthcare. Digital leadership, a leadership approach that utilizes digital technology and information tools to achieve sustainable development, is becoming an important driving force for promoting nurses’ green creativity. Exploring the interaction mechanism between the two is crucial for cultivating nurses’ green creativity.

**Method:**

A cross‐sectional study was conducted on 500 clinical nurses from 24 provinces and 4 municipalities in China using convenience sampling. The Digital Leadership Scale, Innovation Self‐Efficacy Scale, Nurse Organizational Innovation Climate Scale, and Green Creativity Scale were used to measure clinical nurses’ digital leadership, innovation self‐efficacy, nurse organizational innovation atmosphere, and green creativity, respectively. Pearson correlation analysis and structural equation modeling (SEM) were used to analyze the relationships among digital leadership, innovation self‐efficacy, organizational innovation atmosphere, and green creativity.

**Result:**

Digital leadership was found to be a positive predictor of both nurses’ innovation self‐efficacy and organizational innovation atmosphere, with the latter two being significant positive predictors of nurses’ green creativity. Innovative self‐efficacy and organizational innovation atmosphere played a significant mediating role in the relationship between digital leadership and green creativity.

**Conclusion:**

Based on the complex adaptive system, this study reveals that digital leadership significantly promotes nurses’ green creativity through the chain‐mediated pathway of innovative self‐efficacy and organizational innovation atmosphere, which provides a theoretical basis and management strategy for subsequent interventions for nurses’ green creativity by nursing managers in order to synergistically improve the quality of nursing care and sustainable development.

## 1. Background

In response to the real challenges of an aging population, uneven distribution of resources, and environmental pressures, the United Nations established the Sustainable Development Goals in 2025 to provide a framework for implementing environment‐oriented policies [1], and to accelerate the deep integration of sustainable development and digital transformation in the healthcare industry. Sustainable development in the healthcare industry is closely related to nursing work. Nurses, as frontline clinical workers, play a key role in green practices in the healthcare industry. They promote the sustainable development of the entire healthcare system by optimizing resource use, advocating green behavior, participating in the formulation of green policies, and ensuring their own health [2].

The United Nations projects that nearly 75% of the global population will reside in cities by 2050, making sustainable development and public health issues increasingly intertwined [3]. The urbanization process presents opportunities while also posing challenges to the allocation of healthcare resources. Against this backdrop, digital transformation is emerging as a pivotal force reshaping the landscape of healthcare services, propelling nursing practices toward intelligent evolution [4].

As the nursing discipline strives to pioneer sustainable health practices, digital leadership (DL) and green creativity (GC) are increasingly becoming key indicators of nurses’ capacity to address future challenges [5]. Specifically, DL reflects nurses’ ability to integrate technology into nursing practice and drive service innovation within digital environments; GC refers to developing new concepts around green products and practices to address ecological challenges [6]. Although existing research has explored the role of digital transformation in enhancing healthcare efficiency and the potential for nurses to reduce energy consumption through telemedicine and VR technologies [7, 8], systematic studies on the underlying mechanisms linking individual nurses’ DL and GC remain scarce.

As healthcare systems seek sustainable approaches to reduce their environmental footprint while maintaining high‐quality patient care, the concept of GC is becoming increasingly important. Research shows that nurses’ levels of GC vary, but the baseline level is moderate [9]. GC enables nurses to become active promoters of environmental sustainability in healthcare. Through innovative practices and education, nurses contribute to transforming healthcare systems into more sustainable, environmentally friendly, and health‐promoting models [10]. Sustainable nursing practices not only protect the environment but also improve patient outcomes and reduce healthcare costs, helping to improve the quality of care and increase resource efficiency.

Avolio et al. first proposed the concept of DL (e‐leadership), which refers to the social influence process that uses information technology as an intermediary to bring about changes in individuals, teams, and organizations in terms of attitude, emotion, thinking, behavior, and performance [11]. Research shows that DL helps to improve employee innovation behavior, organizational innovation performance, and digital transformation levels [12, 13]. DL leverages tools such as telemedicine, virtual teams, and triage software to enhance care efficiency and quality, enabling rapid and precise service delivery, expanding coverage, and optimizing emergency response. Research by Ismail et al. shows that DL significantly improves nurses’ GC in multiple dimensions: motivation, thinking, behavior, and results [5]. This finding aligns with the research by Acharya et al., who noted that digital platforms empower leaders within healthcare structures to establish online communities, facilitate idea sharing and collective action, engage stakeholders in green initiatives, and drive green innovation [14, 15]. These studies collectively indicate a correlation between DL and GC. However, research exploring the mechanisms underlying DL and GC remains relatively limited at present.

Innovative self‐efficacy (IS) refers to an individual’s confidence in their ability to generate creative ideas and implement innovative solutions [16]. The relationship between self‐efficacy in innovation and GC has been well supported in recent studies, with individuals’ beliefs in their creativity and innovative abilities strongly influencing their ability to generate environmentally sustainable ideas and behaviors. Furthermore, research indicates that nurses with high creative self‐efficacy are more inclined to embrace challenges, experiment with novel approaches, and demonstrate heightened levels of creative dynamism in their nursing practice [17, 18].

According to complex adaptive systems (CAS) theory, healthcare organizations can be conceptualized as networks of interdependent agents whose behaviors and innovations emerge through local interactions within “enabling constraints” set by leadership, rather than through linear top–down control [19]. Within this framework, effective leadership manifests as shaping conditions—information flow, feedback, learning opportunities, and psychological safety—that empower agents (nurses) to self‐organize and adapt, rather than prescribing specific green practices. DL in nursing concretely embodies these enabling constraints: it leverages digital tools and data to enhance collaboration and knowledge sharing and has been empirically demonstrated to stimulate nurses’ GC [5, 20]. These digital conditions correspond to sources of self‐efficacy, thereby enhancing nurses’ IS. IS serves as a clear proximal predictor of green innovation and frequently mediates the relationship between situational factors and creative outcomes in health‐related and environmental domains [16]. Therefore, according to CAS theory, DL can be understood as shaping systemic conditions to transform nurses’ internal adaptive capacity—that is, fostering IS—thereby generating emergent behaviors such as GC.

Therefore, we propose the following Hypothesis H1: IS may mediate the relationship between DL and nurses’ GC.

Organizational innovation atmosphere (OIA) [21] refers to employees’ shared perception of the extent to which the organization supports, encourages, and prioritizes innovation. It reflects the collective view of policies, procedures, practices, and the overall atmosphere that either facilitates or hinders the generation and implementation of new ideas and creative initiatives in the workplace. Basheer Al‐Ghazal et al. [22] showed that encouragement from management and peer support, as well as adequate resources such as time, facilities, and information, are key components in fostering GC. A large‐scale study of 1058 frontline nurses in China’s tertiary hospitals found a strong positive correlation between OIA and nurses’ innovative behaviors, indicating that when nurses perceive a supportive innovation climate, they are more likely to participate in innovative activities [23]. When nurses feel that their green innovation efforts are recognized and rewarded, they are more motivated to participate in green innovation. Organizational innovation fosters an atmosphere that provides employees with flexibility, freedom, and resources to think and act creatively, thereby promoting environmental sustainability [24]. This atmosphere encourages risk‐taking and recognizes green innovation efforts, which directly enhances employees’ GC.

From the perspective of CAS, DL reshapes an organization’s information flows, connectivity, feedback loops, and norms. As an enabling constraint, it encourages exploration and learning rather than compliance and control [5]. Through repeated interactions under these new conditions—including sharing ideas via digital platforms, offering supportive responses, and acknowledging innovative behaviors—nurses collectively developed a shared understanding that the organization supports and values innovation, thereby fostering an organizational climate conducive to innovation [6]. In such an atmosphere, nurses are more likely to self‐organize around environmental challenges, share and restructure ideas, and persist through trial‐and‐error cycles, thereby generating higher levels of GC. Thus, GC is an emergent pattern of individual behavior within an innovation‐oriented atmosphere.

Therefore, we propose Hypothesis H2: OIA may mediate the relationship between DL and nurses’ GC.

The relationship between IS and OIA is positive and mutually reinforcing, a view supported by numerous studies. Research by Le et al. [25] shows that employees with strong IS are more likely to share knowledge, collaborate across teams, and contribute to collective creativity. This behavior helps to build an open and interactive organizational environment that fosters innovation, promotes continuous exchange of ideas, and encourages behavioral support. Fayaz [26, 27] and others have shown that hospitals with a good innovation atmosphere increase nurses’ innovation self‐efficacy by 18%.

From the perspective of CAS, DL—as a higher‐order control factor—shapes the cognitive state and interaction rules of agents (i.e., nurses) by simulating digital and sustainable behaviors. It provides technical and emotional support while configuring collaborative digital platforms. These actions enhance nurses’ IS and strengthen their willingness to experiment with novel eco‐friendly solutions [28]. As self‐efficacy spreads through social interactions, it solidifies into a shared belief—that innovation is supported and expected—ultimately fostering an organizational culture of innovation where green practices become normalized [29]. Therefore, GC emerges as an objective attribute of healthcare systems, reflecting the distributed, self‐organizing adaptive cumulative effect triggered by DL. This effect is sequentially mediated by both IS and OIA.

Consequently, we propose Hypothesis H3: IS and OIA exert a chain‐mediated effect between DL and GC.

Driven by the acceleration of digital transformation in the healthcare industry and the pursuit of sustainable development goals, clinical nurses, as the core practitioners of healthcare services, will play a pivotal role in driving environmentally friendly practices within the healthcare system through their GC. However, existing research has primarily focused on the impact of DL on employee innovation behavior in corporate settings, failing to adequately highlight the significant role of DL in the healthcare sector. Additionally, there has been limited attention on the role of nurses, and to date, no studies have explored the chain‐like transmission pathways between DL and nurses’ GC. Therefore, this study aims to investigate this chain‐like mediating pathway and provide theoretical foundations for healthcare institutions to enhance nurses’ green innovation capabilities through DL.

**FIGURE 1:**
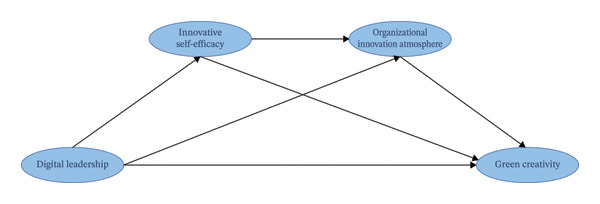
The proposed mediation model.

CAS is a systems science theory proposed by John H. Holland in 1992 [30], considered an important new science for studying complex phenomena in the 21st century. CAS refers to a class of complex systems composed of a large number of adaptive and active individuals (referred to as agents or intelligent entities). These agents form an integrated system through dynamic, nonlinear interactions; the overall behavior exhibits emergent properties and cannot be simply predicted by summing the behaviors of individual agents. CAS was originally used in the scientific field, but research shows that it is still applicable in the medical field and demonstrates its practicality in improving nursing practices [31]. Within an organization, nursing managers, nurses, and the environment form a CAS. Managers apply initial interventions to the system through policies or behaviors, disrupting the original equilibrium. Nurses adjust their actions in response to managerial interventions and, through interaction, spontaneously form new rules or structures, reshaping the organizational environment. This new environment will further regulate nurses’ behaviors through feedback. Based on the CAS framework, this study proposes three hypotheses to construct the hypothetical model as shown in Figure 1.

## 2. Method

### 2.1. Data Collection and Samples

This study employed a cross‐sectional design using convenience sampling. A questionnaire survey was conducted among clinical nurses in 24 provinces and 4 municipalities directly under the central government in China from January 2025 to December 2025. Inclusion criteria were as follows: (1) informed consent and voluntary participation in the study; (2) engaged in clinical nursing work for ≥ 1 year. Exclusion criteria were as follows: (1) interns or trainees in clinical departments; (2) those unable to participate due to travel, training, or other reasons. Based on Hair’s perspective, when a SEM has fewer than seven constructs, the minimum sample size is 300 [32].

This study was conducted by a research team consisting of two nursing professors, one senior nurse, and five nursing graduate students. In accordance with the Declaration of Helsinki, this study was approved by the ethics committee (2024PS1091K). Following the formal authorization from nursing administration leadership, data collection was conducted through the certified online questionnaire survey platform “Questionnaire Star.” Participants received comprehensive digital disclosure regarding study objectives, anonymity guarantees, voluntary participation rights, and data privacy. Technical safeguards were implemented to ensure data completeness and uniqueness: (1) participants can only submit the survey unless they completed all the questions, and (2) the questionnaire allowed only one submission per IP address. Since the data for this study were derived from participant self‐reports, common method bias was effectively controlled through measures such as anonymous completion, concealing variable names, and balancing item sequence effects.

### 2.2. Measures

#### 2.2.1. Sociodemographic Characteristics

Sociodemographic characteristics were compiled by researchers themselves, including 10 items such as gender, age, unit level, and years of service.

#### 2.2.2. DL

This scale was developed by Zhang and Zheng [33] in 2023 and consists of four dimensions: digital thinking transformation ability (5 items), digital resource construction ability (3 items), digital ethics empathy ability (4 items), and digital cognition practice ability (6 items), totaling 18 items. Each item uses a five‐point Likert scale, with scores ranging from “*strongly disagree*” to “*strongly agree*,” assigned values from 1 to 5, respectively. Higher scores indicate stronger DL. Cronbach’s *α* coefficient for this scale is 0.943, and the correlation between each item score and the total scale score is above 0.600. Cronbach’s *α* coefficient for this scale in the present study is 0.904.

#### 2.2.3. GC

The Green Creativity Scale was developed by Jiang et al. [34] in 2021 and consists of 16 items divided into four dimensions: GC motivation (4 items), GC thinking (4 items), GC behavior (4 items), and GC outcomes (4 items). The composite reliability (CR) coefficients for the four latent variables range from 0.76 to 0.80. Each item is scored using a three‐point Likert scale, with values ranging from 1 (*strongly disagree*) to 3 (*strongly agree*). Cronbach’s *α* coefficient for this scale in the present study is 0.847.

#### 2.2.4. IS

This scale was developed by Tierney and Farmer [35] in 2002 as a unidimensional scale, comprising eight items such as “I will be able to achieve most of the goals I set for myself in a creative manner” and “When faced with challenging tasks, I believe I will complete them creatively.” Each item uses a five‐point Likert scale, with scores ranging from 1 (*completely disagree*) to 5 (*completely agree*). Cronbach’s *α* coefficient for this scale in the present study was 0.964.

#### 2.2.5. OIA

This scale was designed by Qian et al. [36] and includes three dimensions: organizational innovation incentives (7 items), resource supply (6 items), and management practices (8 items). Each item uses a five‐point Likert scale, with scores ranging from 1 (*strongly disagree*) to 5 (*strongly agree*). Nurses rate the items based on their own experiences. Higher scores indicate a better OIA. Cronbach’s alpha coefficient for this scale in this study was 0.892.

### 2.3. Data Analysis

Data analysis was conducted using the SPSS 26.0 statistical software package and Amos 28.0. A *p* value of ≤ 0.05 was considered statistically significant. First, the Kolmogorov–Smirnov test was performed to determine normal distribution, and descriptive statistics were used to describe the sociodemographic characteristics of the subjects. Second, Pearson correlation analysis was used to examine the relationships among GC, IS, OIA, and GC. Finally, structural equation modeling was used to test the mediating effects of IS and OIA in the relationship between DL and GC. SEM included two types of variables: latent variables such as DL, IS, OIA, and GC. The four observational variables of DL are digital thinking transformation ability, digital resource construction ability, digital ethics empathy ability, and digital cognition implementation ability. The observational variables of IS are the eight items of the scale. The three observational variables of nurses’ OIA are organizational innovation incentives, resource supply, and management practices. The four observational variables of GC are green creative motivation, green creative thinking, green creative behavior, and green creative outcomes. All SEM analyses were conducted using Amos 28.0. Model fit indices are used to determine the fit of a hypothetical model: root mean square of approximation error (RMSEA), goodness of fit index (GFI), incremental fit index (IFI), comparative fit index (CFI).

## 3. Result

### 3.1. Sociodemographic Characteristics

Table 1 shows the sociodemographic characteristics of the participants. In this study, 84.4% of the participants were female; 46.0% were aged 25–34; 70.0% were from tertiary hospitals; 21.6% had 6–10 years of work experience; 68.6% held a bachelor’s degree. Analysis indicates that nurses’ GC is significantly influenced by the following factors: hospital grade and professional title (*p* < 0.05).

**TABLE 1:** Demographic characteristics and distribution of nurses’ green creativity.

Variant	Options	Frequency	Percentage (%)	F	*p*
Gender	Male	78	15.6	−0.091	0.928
	Female	422	84.4		

Age	< 25	45	9.0	0.009	0.993
	25–34	230	46.0		
	35–44	179	35.8		
	≥ 45	46	9.2		

Hospital level	Level I hospitals	28	5.6	2.023	0.044
	Level II hospitals	122	24.4		
	Tertiary hospital	350	70.0		

Years of working experience	1–5 years	156	31.2	−0.674	0.501
	6–10 years	108	21.6		
	11–15 years	112	22.4		
	≥ 16 years	124	24.8		

Academic qualifications	College and below	43	8.6	0.359	0.72
	Undergraduate	343	68.6		
	Master’s degree and above	114	22.8		

Professional title	Nurse	78	15.6	2.262	0.024
	Nurse assistant	162	32.4		
	Head nurse	192	38.4		
	Associate nurse practitioner and above	68	13.6		

Marital status	Unmarried	177	35.4	0.211	0.833
	Married	315	63.0		
	Else	8	1.6		

Experience in teaching or not	Yes	326	65.2	0.682	0.496
	No	174	34.8		

Working section	General medicine	194	38.8	−0.349	0.727
	Neurosurgery	151	30.2		
	Gynecology	22	4.4		
	Intensive care unit	62	12.4		
	Operating rooms	34	6.8		
	Emergency call	23	4.6		
	Department of gynecology and obstetrics	14	2.8		

Nature of employment	Staff nurses	222	44.4	0.894	0.372
	Nonstaff nurses	278	55.6		

### 3.2. Common Method Bias Test

Harman’s one‐factor test identified 12 factors with eigenvalues greater than 1 without rotation. The first factor explained 27.972% of the variance, below the 40% critical threshold, indicating that common method bias had no significant impact on the study [37].

### 3.3. Examination of Multicollinearity

The data assumptions were checked to ensure the reliability of the study. Skewness coefficients for all variables were found to range between −0.966 and −0.334, while the kurtosis coefficients ranged between −0.283 and 1.214. Consistent with Kline’s (2016) standards, these values confirm a normal data distribution. Kline noted that when skewness falls between ±3 and kurtosis between ±10, data do not significantly deviate from a normal distribution [38]. The variance inflation factor (VIF) was observed to vary between 1.053 and 3.724, well below the recommended threshold of 5 proposed by Hair et al. [32]. The study demonstrated that there were no issues related to multicollinearity or residuals.

### 3.4. Correlation Analysis

In this analysis, Pearson correlation analysis was used to explore the relationships between the variables. The results show that there are significant correlations between all variables in this analysis (*p* < 0.01). Based on the correlation coefficients, all variables have *r > 0*, indicating that there are significant positive correlations between all variables in this analysis, as shown in Table 2.

**TABLE 2:** Results of Pearson correlation analysis between variables (*n* = 500).

Variables	DL	IS	OIA	GC
DL	1			
IS	0.595^∗∗^	1		
OIA	0.576^∗∗^	0.564^∗∗^	1	
GC	0.451^∗∗^	0.570^∗∗^	0.517^∗∗^	1

Abbreviations: DL, digital leadership; GC, green creativity; IS, innovative self‐efficacy; OIA, organizational innovation atmosphere.

^∗∗^Significance at *p* < 0.001.

### 3.5. Measurement Model

The measurement model was estimated using maximum likelihood estimation to evaluate item reliability, model convergent validity, and discriminant validity.

#### 3.5.1. Convergent Validity

As shown in Table 3, no item had factor loadings below 0.6; therefore, none required exclusion [39]. Item factor loadings ranged from 0.654 to 0.921. CR for each construct ranged from 0.764 to 0.980, with average variance extracted (AVE) fluctuating between 0.520 and 0.771. All measurement model indices met the standards for model reliability and convergent validity.

**TABLE 3:** Factor loading of confirmatory factor analysis (CFA).

Latent variables	Measurement variable	Factor loading	CR	AVE
DL	DL1	0.724	0.844	0.575
	DL2	0.781		
	DL3	0.767		
	DL4	0.759		

IS	IS1	0.823	0.964	0.771
	IS2	0.853		
	IS3	0.869		
	IS4	0.874		
	IS5	0.911		
	IS6	0.921		
	IS7	0.892		
	IS8	0.878	0.765	0.549

OIA	OIA1	0.683		
	OIA2	0.720		
	OIA3	0.759		

GC	GC1	0.654	0.764	0.520
	GC2	0.685		
	GC3	0.676		
	GC4	0.664		

Abbreviations: DL, digital leadership; GC, green creativity; IS, innovative self‐efficacy; OIA, organizational innovation atmosphere.

#### 3.5.2. Discriminant Validity

Discriminant validity was assessed using Fornell and Larcker’s method [40]. When the square root of a construct’s AVE surpasses its correlations with other constructs, discriminant validity is established. In this study, the square root of the AVE for each latent variable exceeds the corresponding correlation coefficient in its row and column, indicating that each construct is effectively correlated while maintaining independent distinctiveness, confirming the discriminant validity among scales (refer to Table 4 for more information).

**TABLE 4:** Results of discriminant validity by AVE.

Variables	DL	IS	GC	OIA
DL	**0.758**			
IS	0.595	**0.878**		
GC	0.451	0.570	**0.670**	
OLA	0.576	0.564	0.517	**0.721**

*Note:* The bold values represent the square roots of the AVE values for each construct.

Abbreviations: DL, digital leadership; GC, green creativity; IS, innovative self‐efficacy; OIA, organizational innovation atmosphere.

### 3.6. Mediation Effect Analysis

**FIGURE 2:**
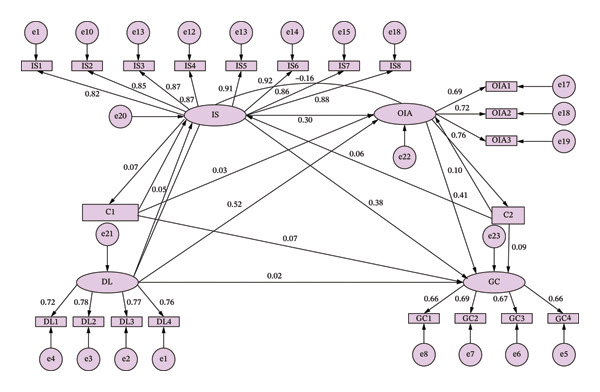
SEM of digital leadership on the pathway structure of nurses’ green creativity. DL: digital leadership; IS: innovative self‐efficacy; OIA: organizational innovation atmosphere; GC: green creativity; C1: hospital level; C2: professional title.

The SEM was tested in AMOS 28.0 software. Variables showing statistical significance in the demographic data were used as control variables. DL served as the independent variable, GC as the dependent variable, and innovation self‐efficacy and organizational innovation climate as mediating variables to construct the SEM. Model estimation employed the maximum likelihood method. Table 5 indicates good model fit, while Figure 2 displays the standardized estimates of model parameters.

**TABLE 5:** SEM model fit results for the path of digital leadership’s effect on green creativity.

Items	Reference standard	Actual results
CMIN/DF	1–3 is excellent, 3–5 is good	3.348
RESMA	< 0.05 is excellent, < 0.08 is good	0.069
IFI	> 0.9 is excellent, > 0.8 is good	0.970
TLI	> 0.9 is excellent, > 0.8 is good	0.964
CFI	> 0.9 is excellent, > 0.8 is good	0.969

*Note:* CMIN/DF = chi‐square.

Abbreviations: CFI, comparative fit index; IFI, incremental fit index; RMSEA, root mean square error of approximation; TLI, Tucker–Lewis index.

The mediation effects were tested using the bootstrap technique, a statistical method that constructs empirical distributions through repeated sampling to estimate the uncertainty of statistical parameters [41]. In this study, the 95% confidence intervals of each mediation effect were derived via 5000 bootstrap samples, in which the significance of the mediating effect is determined based on whether the confidence interval includes zero.

Research findings indicate that DL significantly predicts nurses’ GC, with a total effect size of 0.214. The relationship between DL and nurses’ green creativity is mediated by IS and OIA, with effect sizes of 0.094 and 0.080, respectively, accounting for 46.07% and 39.21% of the total indirect effect. Furthermore, the chained mediation effect controlled for innovation self‐efficacy and OIA revealed that the mediation effect of DL on nurses’ GC was 0.080, accounting for 15.20% of the total indirect effect (Table 6).

**TABLE 6:** Bootstrap mediation effect test results.

Action path	*β*	SE	LLCI	ULCL	*p*	Effect proportion (%)
Total effect	0.214	0.089	0.164	0.272	< 0.001	
Direct effects	0.009	0.071	−0.067	0.081	0.810	
Indirect effect	0.204	0.109	0.145	0.285	< 0.001	
DL ⟶ IS ⟶ GC	0.094	0.028	0.052	0.141	< 0.001	46.07
DL ⟶ OIA ⟶ GC	0.080	0.096	0.033	0.146	0.001	39.21
DL ⟶ IS ⟶ OIA ⟶ GC	0.031	0.032	0.013	0.060	< 0.001	15.20

Abbreviations: DL, digital leadership; GC, green creativity; IS, innovative self‐efficacy; OIA, organizational innovation atmosphere.

Notably, when considering the mediation effect, the direct impact of DL on nurses’ GC was no longer significant. This supports the view that DL primarily influences nurses’ GC through the proposed mediating mechanisms, rather than through direct pathways. We therefore conclude that our data support the proposed mediating mechanism, while recognizing that a nonsignificant total/direct effect cautions against claiming a strong overall impact of DL on nurses’ GC and does not rule out the existence of additional, unmodeled mediators.

## 4. Discussion

As healthcare systems face increasingly serious environmental issues such as climate change and resource depletion, the concept of GC is becoming increasingly important. Nurses, as frontline healthcare providers, can lead sustainable change by integrating ecological awareness into clinical practice and collaborating with other professionals to develop sustainable healthcare systems. This approach minimizes the environmental impact of healthcare delivery while improving patient care quality and resource utilization efficiency [9]. This study indicates that DL, IS, and OIA are all positively correlated with GC. In particular, IS and OIA play partial mediating and sequential mediating roles in the relationship between DL and GC. High‐level DL correlates with clinical nurses’ high IS and influences the formation of OIA, thereby further affecting nurses’ levels of GC. This reveals the potential mechanism through which DL impacts GC.

### 4.1. The Mediating Effect of IS Between DL and GC

IS plays a significant mediating role between DL and GC, which is consistent with research hypothesis H1. As per CAS theory, DL shapes organizations into CAS by creating digital and flexible conditions, thereby enhancing employees’ IS. This heightened self‐belief enables GC to emerge through self‐organizing, adaptive interactions within the system. DL provides digital tools, information, and collaborative platforms to promote learning and innovation related to environmental challenges [42]. Through DL, clinical nurses develop their IS, which is enhanced by digital leaders who provide training, resources, recognition, and a psychologically safe environment. Higher levels of IS are related to higher levels of nurses’ GC. When individuals are confident in their innovative abilities, they are more motivated and capable of generating sustainable innovations that benefit the organization and the environment, which is consistent with the research findings of Henriques et al. [43]. Research by Meirun et al. [44] shows that clinical nurses with high IS are 68% faster than others in adopting green anesthetic gas capture systems because they believe in their ability to solve technical challenges. Effective use of digital tools can enable clinical nurses to believe in their innovative potential, thereby generating more green creative outcomes and promoting sustainable development in the healthcare industry.

### 4.2. The Mediating Effect of OIA Between DL and GC

Organizational innovation fosters a supportive work environment that encourages nurses to generate and implement innovative green ideas, thereby mediating between DL and GC, which is consistent with research hypothesis H2. DL shapes the organization as a CAS by establishing a digital, flexible innovation climate, and this system‐level climate then enables GC to emerge as employees self‐organize, interact, and adapt to generate and implement environmentally friendly ideas. Research shows [45, 46] that AI‐powered mentor platforms connecting nurses with environmental engineers and supply chain experts increased green patent applications by 214% in a multicenter trial. These digital platforms eliminate barriers, enabling nurses to integrate sustainability principles into clinical workflows through interdisciplinary solution design. In this environment, green innovation will receive greater attention, support, and rewards. Although most existing research [22] has focused on green transformational leadership, these principles are equally applicable to DL, as digital leaders enhance the innovation environment by leveraging technology to associate with collaboration, knowledge sharing, and resource accessibility—all of which are critical to GC. Therefore, DL indirectly influences GC by fostering an organizational innovation environment that empowers employees to explore and implement environmentally sustainable innovations. This mediating role underscores the importance of establishing a culture and environment conducive to green innovation under the influence of DL.

### 4.3. The Chain‐Mediated Effect of IS and OIA Between DL and GC

The relationship between DL and GC is not a simple direct connection but rather a gradual transmission through cognitive and cultural levels. Specifically, DL first may contribute to the enhancement of nurses’ IS, and the general enhancement of nurses’ IS in turn is conducive to the formation of a good OIA in the medical system. In this cultural environment, the GC of the nursing group is significantly enhanced. This is consistent with the hypothesis H3 of this study. Research shows [42] that digital tools such as AR simulations provide an excellent environment for skill acquisition. Nurses who participated in VR‐based sustainability training programs demonstrated a 73% higher increase in IS than their peers. Nurses with high IS help to create a positive OIA. In such an atmosphere, nurses are more likely to design multifaceted, creative solutions to problems. A study of 1058 Chinese nurses during the Omicron pandemic showed [47] that a good OIA increased nurses’ GC by 41.7%. Similarly, research findings by Ismail et al. [5] show that hospitals with dedicated innovation centers reported higher levels of nurse involvement in the design of energy‐efficient infrastructure.

In addition, certain measures are still needed to utilize this intermediary path to transform the digital strategy in the healthcare system into green innovation outcomes. For example, integrating DL into the curriculum to provide nurse leaders with data analysis and ecological innovation capabilities; research shows that establishing green innovation indicators [9], i.e., tracking outcomes such as energy savings and waste reduction in performance evaluations, will also promote this transformation.

## 5. Conclusion

In the evolving healthcare field, DL has emerged as a key force in driving sustainable innovation, particularly in promoting GC—i.e., the development of environmentally friendly solutions in nursing practice. This study demonstrates that integrating DL training and practice, strengthening the construction of nurses’ innovation self‐efficacy, and creating a positive organizational climate for innovation are effective pathways for enhancing nursing GC and promoting sustainable healthcare. Healthcare organizations and policymakers should emphasize the value of DL in nursing green innovation and promote the implementation of DL education and sustainability management practices, thereby facilitating nursing teams to achieve both environmental and service breakthroughs in the digital era.

## 6. Limitations

Firstly, this study relied heavily on questionnaires, which may be subject to self‐report bias. Future research could consider using more diverse data collection methods such as experiments, interviews, or case studies. Secondly, the sample primarily originates from a single country, China. This limitation may restrict the generalizability of the research outcomes. Future investigations should broaden the sample scope to examine the validity of these findings across different geographical regions within China and incorporate more diverse cultural and economic contexts. Finally, this study mainly focuses on the chain‐mediated mechanism of innovation self‐efficacy and organizational innovation climate in the relationship between DL and GC and does not further explore the role of the corresponding moderating variables; there may be other important variables that deserve attention, and future research could try to examine the contextual effects of other environmental factors in this process in order to deepen the findings of this study.

## Funding

This research did not receive any specific grant from funding agencies in the public, commercial, or not‐for‐profit sectors.

## Conflicts of Interest

The authors declare no conflicts of interest.

## Data Availability

The data that support the findings of this study are available from the corresponding author upon reasonable request.
